# EEC-LM-ADULT syndrome caused by R319H mutation in TP63 with ectrodactyly, syndactyly, and teeth anomaly: A case report

**DOI:** 10.1097/MD.0000000000022816

**Published:** 2020-10-30

**Authors:** Yuki Otsuki, Koichi Ueda, Takashi Nuri, Chisei Satoh, Ryuta Maekawa, Koh-ichiro Yoshiura

**Affiliations:** a Department of Plastic and Reconstructive Surgery, Osaka Medical College, Osaka; b Department of Human Genetics, Atomic Bomb Disease Institute, Nagasaki University Graduate School of Biomedical Sciences, Nagasaki, Japan.

**Keywords:** case report, ectrodactyly, EEC/LM/ADULT syndrome, tooth shape abnormalities, TP63

## Abstract

**Rationale:**

Ectrodactyly ectodermal dysplasia-cleft lip/palate (EEC) syndrome, limb-mammary syndrome (LMS), and acro-dermato-ungual-lacrimal-tooth (ADULT) syndrome are caused by a *TP63* gene disorder and have similar features. In the present article, a R319H mutation in TP63 is reported, and the correlation between genotype and phenotype is discussed based on the current case and previous literature.

**Patient concerns:**

A 13-year-old Japanese boy had ectrodactyly in the right hand and left foot and syndactyly in the left and right foot, and tooth shape abnormalities.

**Diagnoses:**

Peripheral blood samples were obtained, and mutation analysis was performed. A heterozygous G>A transition at cDNA position 956 of the TP63 gene was found. The patient was diagnosed with ELA (EEC/LM/ADULT) syndrome based on his clinical features and mutation analysis results.

**Interventions:**

The patient underwent surgery to correct the left foot malformation at 1 year of age and the right foot syndactyly at 11 years of age.

**Outcomes:**

No complications were observed after the first and second operations. He can walk comfortably after them, and no additional interventions will be planned in him. We continued to follow up with him up to the present.

**Lessons:**

The concept of ELA syndrome, which is the original concept of combining 3 syndromes (EEC syndrome/LMS/ADULT syndrome) into a unique clinical entity, can help clinicians to better understand TP63-related syndromes and improve the differential diagnosis of these syndromes.

## 1 Introduction

Ectrodactyly or split hand/foot malformation is a rare congenital limb disorder with a prevalence of approximately 1 in 18,000 newborns.^[1]^ TP63, a member of the p53 gene family and a major tumor suppressor gene, is highly expressed in embryonic ectoderm tissues in adults.^[2]^ TP63 acts as a key regulator in limb development and other tissues, including epithelial and craniofacial tissues, not classical tumor-suppressor genes.^[3]^ Hence, ectrodactyly sometimes occurs along with other symptoms.

Causative *TP63* mutations have been identified in 6 different syndromes: ectrodactyly ectodermal dysplasia-cleft lip/palate (EEC) syndrome, acro-dermato-ungual-lacrimal-tooth (ADULT) syndrome, limb-mammary syndrome (LMS), ankyloblepharon-ectodermal dysplasia-clefting (AEC) syndrome, Rapp–Hodgkin syndrome (RHS), and nonsyndromic split-hand/split-foot malformation (SHFM). EEC syndrome, LMS, and ADULT syndrome have traits along the same clinical spectrum and a similar pattern of p63 mutations; thus, the concept of ELA (EEC-ADULT-LM) syndrome has been reported to simplify the classification of TP63-associated disorders.^[4]^ In the present article, a case of R319H (described as R280H) mutation in TP63 with ectrodactyly, syndactyly, and tooth anomaly, is reported, and the correlation between genotype and phenotype is discussed based on the current case and previous literature.

## 2 Case presentation

**Figure 1:**
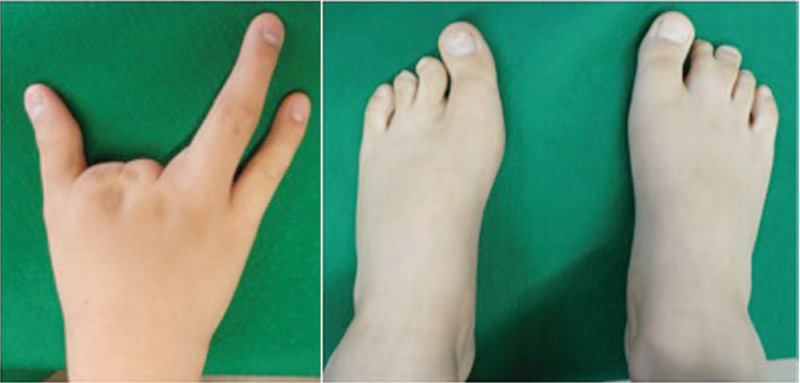
The hand and foot disorders in the patient. Hand and foot disorders in the present patient at 10 years of age. Clinical photographs show split hand and foot malformations.

**Figure 2:**
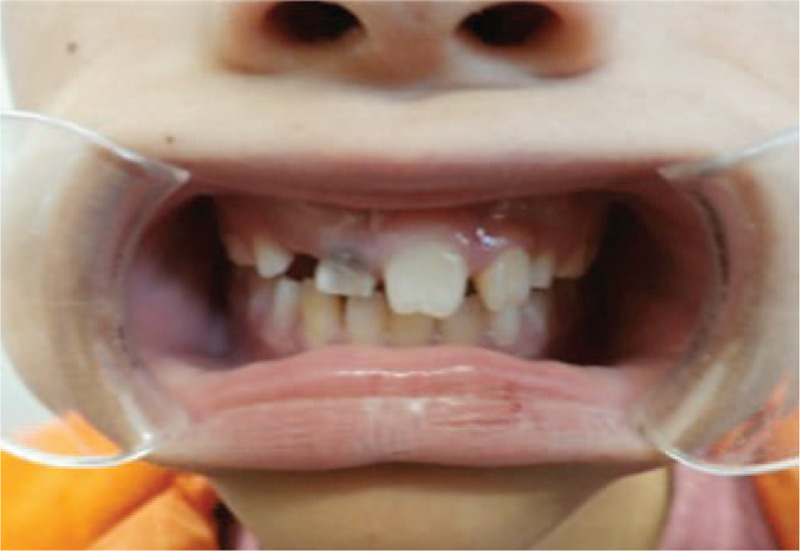
The patient's teeth at 13 years of age. The patient's teeth were 13 years old. Intraoral photographs show peg-shaped teeth.

The patient was a 13-year-old Japanese boy who was delivered normally, weighing 2954 g. He was found to have ectrodactyly in the right hand and left foot (Fig. 1), with syndactyly in the left and right foot. He underwent surgery to correct the left foot malformation at 1 year of age and the right foot syndactyly at 11 years of age. No complications were observed. He can walk comfortably after them, and no additional interventions will be planned in him. Furthermore, the patient had skin disorders involving dry skin and café au lait spots as well as tooth shape abnormalities in which the edge of the incisors was notched and narrower than the cervical area, giving a peg-shaped appearance (Fig. 2). Cleft lip and palate, mammary gland hypoplasia, lachrymal duct atresia, and ankyloblepharon were not observed. The patient also had attention deficit hyperactivity disorder (ADHD), which was not thought to be related to the disorder. Neither the patient nor his parents had a history of carcinomas.

**Figure 3:**
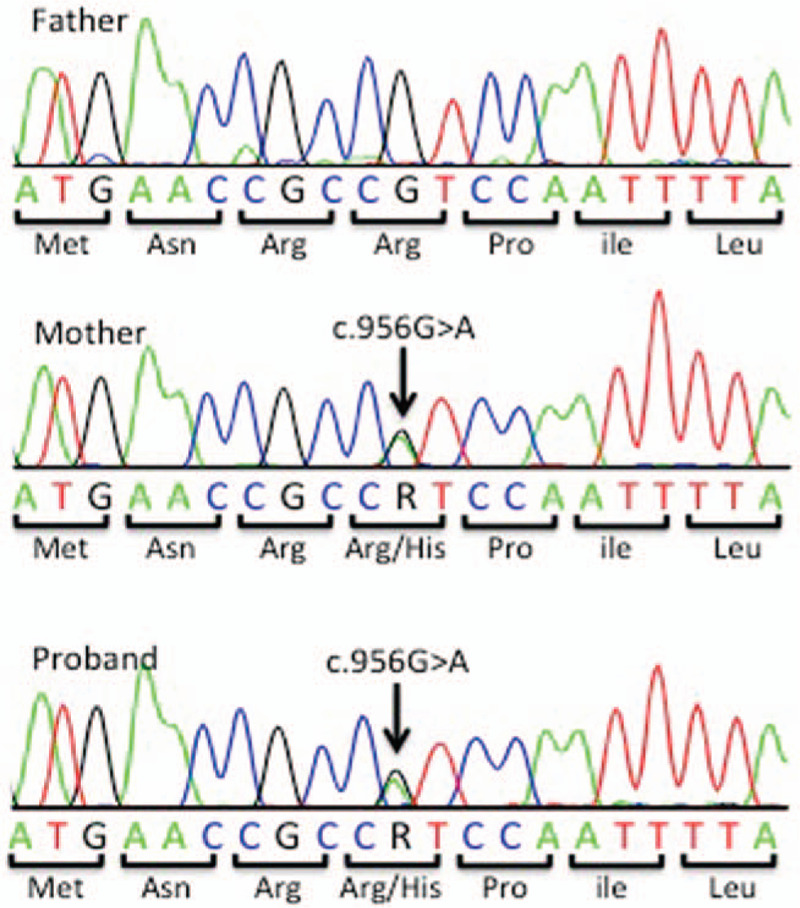
p63 mutation analysis. *TP63* mutation analysis. A heterozygous G>A transition at cDNA position 956 of the *TP63* gene was found in the patient, and the same mutation was also detected in his mother.

After ethical approval by the ethics committee of Osaka Medical College (approval number 2772) and written informed consent from the patient and his parents, peripheral blood samples were obtained. Whole exome sequencing was performed to screen for candidate causative mutations. Called mutations were validated by Sanger sequencing. The results showed no de novo, homozygous, and compound heterozygous mutations. Next, deleterious mutations (nonsynonymous, frameshift, or splice site mutations) with a frequency <0.5% in the general population of p63 that were transmitted from one of the parents were analyzed, and a maternally transmitted missense mutation (c.956G>A, p.(R319H), ENST00000264731) was found (Fig. 3). This mutation was previously reported as a de novo mutation (referred to as R280H) in 2 patients with SHFM or EEC syndrome, and mutations in the same locus have been reported in 9 patients, including our case.^[3]^ Therefore, the patient was diagnosed with ELA (EEC/LM/ADULT) syndrome based on his clinical features and mutation analysis results. His mother showed the same missense mutation at nucleotide 956, but she did not have any abnormalities related to the missense mutation. All relevant information was explained to the patient and his mother, and written informed consent was obtained from the patient and his mother for publication of this case report and accompanying images.

## 3 Discussion

TP63 is usually expressed in epithelial and mesenchymal structures; thus, TP63 mutations cause disorders in these tissues. At least 6 different syndromes have been linked to *TP63* mutations, including EEC syndrome, ADULT syndrome, LMS, AEC syndrome, RHS, and SHFM. Of these, AEC and RHS represent a clinical spectrum that differs from the other 4 syndromes^[1,4]^ because of the lack of ectrodactyly, and they are associated with different patterns of *TP63* gene mutations^[5]^ (Table 1). However, the remaining 4 syndromes have several overlapping manifestations, rendering differential diagnosis difficult.^[6]^ The main symptoms of EEC syndrome are ectrodactyly, ectodermal dysplasia, and cleft lip/palate, but ectrodactyly is also frequently present in ADULT syndrome and LMS. Furthermore, ectodermal dysplasia is present in all *TP63* mutation syndromes. Thus, some reports showed an intermediate phenotype between EEC syndrome and ADULT syndrome.^[1,4]^ To distinguish them, the presence of cleft lip and palate has been proposed as a critical feature. However, only two-fifths of patients presented with a cleft lip or palate in EEC syndrome.^[7,8]^ Therefore, this feature is not definitive for EEC syndrome. The present case showed ectrodactyly, syndactyly, tooth disorder, and skin lesions. The absence of cleft lip or palate with ectrodactyly and ectodermal dysplasia can support a diagnosis of ADULT syndrome, but the lack of nail and lachrymal duct symptoms probably undermines the rationale for the diagnosis. Furthermore, R319H (described as R280H previously) is the causative gene mutation in the present case, and it has been reported in both EEC syndrome and SHFM. This mutation also helps to rule out ADULT syndrome. For such cases, family history can aid in the diagnosis. In the present case, the patient's mother had the same missense mutation, but she did not have any symptoms or signs. The reason why she had no manifestations of the disease despite her missense mutation is unclear, but it may be related to somatic mosaicism^[9]^ or variable penetrance.

**Table 1:**
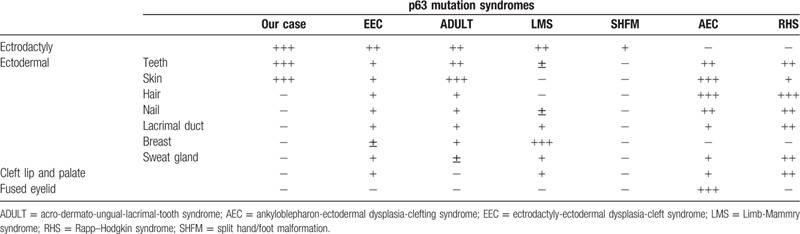
Contrast phenotypic characteristics of our case and p63 mutation syndromes.

The concept of EEC syndrome was established before the detailed determination of its genetic causes. Therefore, EEC syndrome is defined by a combination of phenotypes.^[10]^ However, the phenotype exists based on the genotype, so that the genotype–phenotype correlation cannot be ignored when the origins of the syndromes are elucidated. This is the cause of confusion in understanding TP63-related syndromes. Prontera et al^[4]^ suggested the original concept of combining these syndromes into a unique clinical entity, ELA (EEC/LM/ADULT) syndrome. Establishment of a better classification system based on genotype–phenotype correlation can help clinicians to better understand TP63-related syndromes and improve the differential diagnosis of these syndromes.

## Acknowledgments

The funders had no role in study design, data collection and analysis, decision to publish, or preparation of the manuscript.

## Author contributions

**Conceptualization:** Koichi Ueda.

**Data curation:** Chisei Satoh, Ryuta Maekawa, Koh-ichiro Yoshiura.

**Methodology:** Koichi Ueda, Koh-ichiro Yoshiura.

**Project administration:** Koichi Ueda.

**Supervision:** Koichi Ueda.

**Validation:** Takashi Nuri.

**Visualization:** Yuki Otsuki.

**Writing – original draft:** Yuki Otsuki.

**Writing – review & editing:** Yuki Otsuki, Chisei Satoh.
